# Combinatory annotation of cell membrane receptors and signalling pathways of *Bombyx mori* prothoracic glands

**DOI:** 10.1038/sdata.2016.73

**Published:** 2016-08-30

**Authors:** Panagiotis Moulos, Martina Samiotaki, George Panayotou, Skarlatos G. Dedos

**Affiliations:** 1 HybridStat Predictive Analytics, Aiolou 19, Athens 10551, Greece; 2 Biomedical Sciences Research Center ‘Alexander Fleming’, Fleming 34, Vari 16672, Greece; 3 Department of Biology, National and Kapodistrian University of Athens, Athens 15784, Greece

**Keywords:** Cellular signalling networks, Cell signalling

## Abstract

The cells of prothoracic glands (PG) are the main site of synthesis and secretion of ecdysteroids, the biochemical products of cholesterol conversion to steroids that shape the morphogenic development of insects. Despite the availability of genome sequences from several insect species and the extensive knowledge of certain signalling pathways that underpin ecdysteroidogenesis, the spectrum of signalling molecules and ecdysteroidogenic cascades is still not fully comprehensive. To fill this gap and obtain the complete list of cell membrane receptors expressed in PG cells, we used combinatory bioinformatic, proteomic and transcriptomic analysis and quantitative PCR to annotate and determine the expression profiles of genes identified as putative cell membrane receptors of the model insect species, *Bombyx mori*, and subsequently enrich the repertoire of signalling pathways that are present in its PG cells. The genome annotation dataset we report here highlights modules and pathways that may be directly involved in ecdysteroidogenesis and aims to disseminate data and assist other researchers in the discovery of the role of such receptors and their ligands.

## Background & Summary

Insect cell membrane receptors are excellent drug targets for pest control and management^1,2^. As such, an extensive amount of research has been carried out on various cell membrane receptors expressed in model insects, such as *Drosophila melanogaster*^3^ and *Bombyx mori*^4,5^, or insects that are major pests of crops or agents of animal and human diseases^6–9^. Identifying species-specific receptors expressed in cells crucial for the development of insects, such as the prothoracic gland (PG) cells, is even more important as these receptors can become targets for highly specialised, precise and species-specific drug discovery.

The PG cells are the main site of synthesis of ecdysteroids from cholesterol and their subsequent secretion^10,11^. Research efforts have shown that the signalling pathways that govern ecdysteroidogenesis are quite complex and involve a very broad array of second messengers and signalling modules with a high degree of overlapping integration and redundancy, albeit a large number of unidentified ligands, receptors and signalling components^10,12–16^. Much work on the ligands and receptors that stimulate or inhibit ecdysteroidogenesis has been carried out in model insects such as *Bombyx mori*^12,14,17^, whose extensive genome annotation is publicly available with genes mapped to chromosome and scaffold locations and the sequences of their coding protein(s)^18,19^.

**Figure 1:**
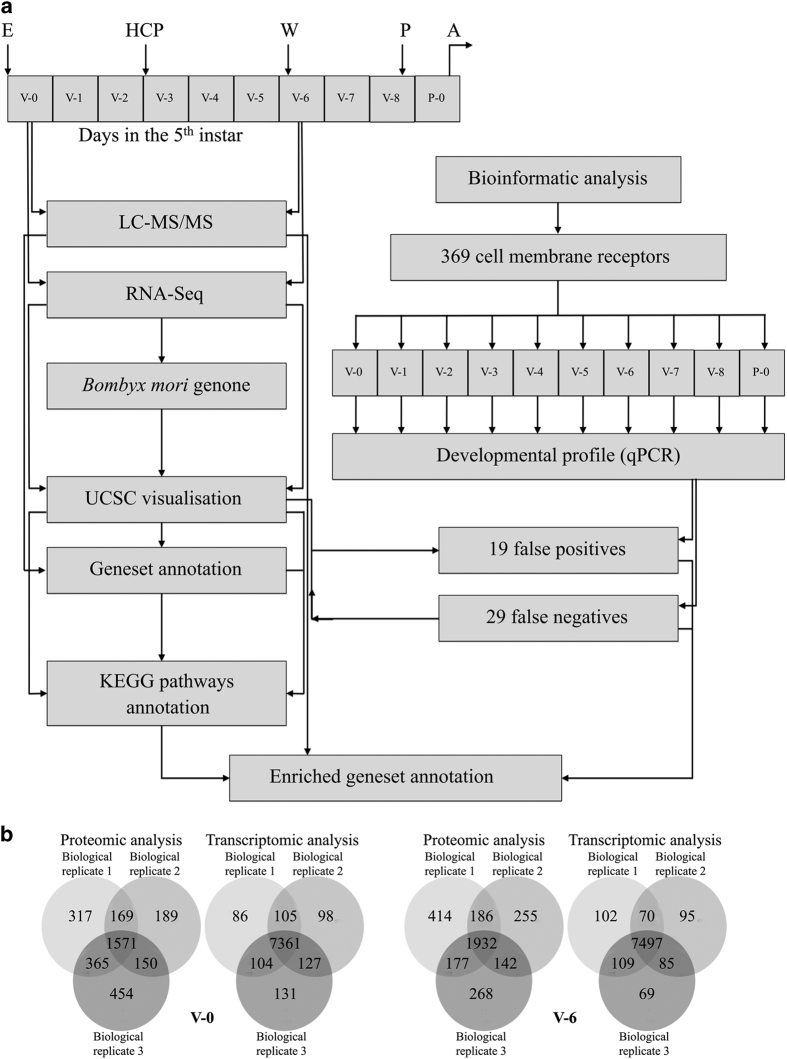
Schematic workflow of the experimental design. **a**: The figure shows the iterative approaches we followed to identify the cell membrane receptors expressed in prothoracic gland cells of *Bombyx mori*. False positive and false negative hits based on the liquid chromatography-tandem mass spectrometry (LC-MS/MS) and transcriptomic (RNA-seq) analyses were analysed by qPCR assays and further examined by visualization in the University of California, Santa Cruz (UCSC) Genome Browser. Abbreviations indicate the time of ecdysis (E) to the final larval stage, the time of head critical period (HCP; see text for details), the time of feeding cessation and onset of wandering (W) behaviour, the time of metamorphosis to pupa (P) and the time of initiation of apoptosis (A) by the prothoracic gland cells. **b**: Venn diagrams showing the results of liquid chromatography-tandem mass spectrometry (LC-MS/MS) and transcriptomic (RNA-seq) analyses of the 3 biological replicates from day 0 (V−0) and day 6 (V−6) from prothoracic gland cells of the 5th instar of *Bombyx mori*.

**Figure 2:**
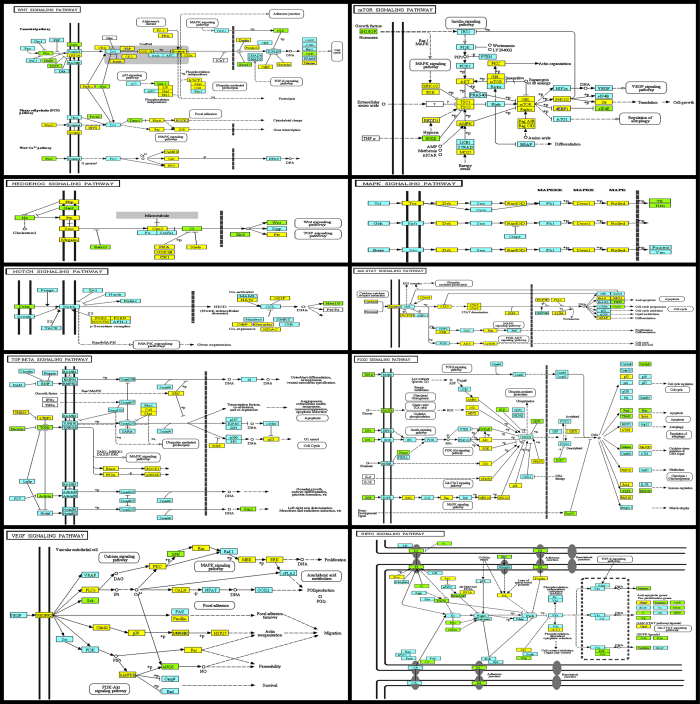
KEGG database-adopted signalling pathways identified in the prothoracic gland cells of the silkworm, *Bombyx mori*. Signalling pathways from KEGG database were manually edited to highlight the signalling cascade components i) identified by our proteomic and transcriptomic data (yellow boxes), ii) identified by our transcriptomic (and qPCR analyses for the cell membrane receptors) but not by our proteomic data analyses (blue boxes), iii) not identified by both our proteomic and transcriptomic data analyses (green boxes) to be expressed by the prothoracic gland cells of the silkworm, *Bombyx mori*. White boxes indicate signalling cascade components not identified in the genome of the silkworm, *Bombyx mori*. Readers are referred to the KEGG database and Data Citation 1 for detailed description of the abbreviated terms.

In this study we further annotated the existing dataset of *Bombyx mori* genes^18,19^ (http://sgp.dna.affrc.go.jp/ComprehensiveGeneSet/) to include 1) genes expressed in the PG cells identified by our proteomic (using liquid chromatography-tandem mass spectrometry (LC-MS/MS)) and transcriptomic (RNA-seq) datasets (Fig. 1) as described in a supporting paper^20^, 2) annotations of signalling pathways that are present in PG cells (Fig. 2), 3) annotations of *Bombyx mori* genes with the LocTree3^21^ protein subcellular localisation prediction dataset (Data Citation 1) and 4) annotation of the coding sequences of the proteins (Data Citation 1), by integrating results of our bioinformatics, LC-MS/MS and RNA-seq analyses from PG samples from day 0 (V−0) and day 6 (V−6) of the 5th instar (Data Citation 1) and further analysis of the expression patterns of these receptors by quantitative PCR (qPCR).

To implement our workflow (Fig. 1a), first we generated experimental data and analysis of LC-MS/MS datasets coupled with experimental data and analysis of RNA-seq datasets (Fig. 1 and Data Citation 1,Data Citation 2 and Data Citation 3). We next created a *Bombyx mori* reference genome dataset (Data Citation 4) to map and visualise the RNA-seq data and, in parallel, we generated literature-based and bioinformatics-assisted annotated lists of cell membrane receptors present in the genome of *Bombyx mori*. Next, we validated the expression of cell membrane receptors in PG cells through qPCR and clarified the presence of false negative and false positive hits in our LC-MS/MS and/or RNA-seq datasets (Fig. 1a and Tables 1 and 2). Critical to our approach was the visualization of RNA-seq data through the University of California, Santa Cruz (UCSC) Genome Browser^22^ track data hubs webpage, hosting the *Bombyx mori* reference genome (Data Citation 4), that allowed us to explain false positive and false negative hits (Tables 1 and 2) in our LC-MS/MS, RNA-seq and qPCR data.

All these datasets were integrated back into i) the annotated list of *Bombyx mori* genes that is publicly available (Fig. 1 and Data Citation 1) ii) the publicly available KEGG^23^ signalling pathways of *Bombyx mori* that we further annotated, visualized and present in Fig. 2 iii) the UCSC Genome Browser^22^ track data hubs webpage where results of the RNA-Seq samples analyses can be visualised by pasting the following link (http://epigenomics.fleming.gr/tracks/hs_trackhubs/ekpa_dedos_2/hub.txt) and then pasting the coordinates of a gene of interest.

We provide a substantially increased list of *Bombyx mori* annotated genes (Data Citation 1) integrated with our LC-MS/MS and RNA-seq datasets (Fig. 1b) and other bioinformatic annotations of *Bombyx mori* genes (http://epigenomics.fleming.gr/metaseqr_runs/HS/000004/) and most importantly we identify and visually depict here (Fig. 2) the signalling pathways in PG cells reported in a supporting paper^20^. Our datasets can be valuable tools for researchers who want to study the role of signalling pathways in PG cells or conduct comparative studies on the presence of cell membrane receptors in other insect species. Additional information for a comprehensive understanding of the cell membrane receptors and the signalling pathways in PG cells is described in a related research paper^20^.

## Methods

### Animals

The hybrid J106xDAIZO of *Bombyx mori* was used in this study. In this hybrid, the 5th instar period lasts about ~208 h, the onset of pupal commitment occurs after 60 h (day 3) and the onset of wandering behaviour occurs 144 h (day 6) after the final larval ecdysis. This hybrid has a short period of cocoon spinning that lasts ~38 h followed by a period of ~26 h before pupal metamorphosis. In this study, each day of the 5th (V) instar is designated with its numerical number (i.e., V−0, V−1 etc.) while the first day of the pupal stage is designated as P−0. Larvae were reared on fresh mulberry leaves under a 12:12-L:D photoperiod at 25±1 °C and 60% relative humidity. Larvae were staged after every larval ecdysis, and the day of each ecdysis was designated as day 0. Since larvae mainly moult to the final (5th) instar during the scotophase, all larvae that ecdysed during the scotophase were segregated immediately after the onset of photophase. This time was designated as 0 h of the 5th instar and 4 h later samples of prothoracic glands were taken (day 0 samples) while samples of prothoracic glands from day 6 were taken 144 h later, at the onset of wandering behaviour.

### Experimental design

The aim of our present study was to provide a thorough map of signalling cascades present in the PG cells of *Bombyx mori* during the crucial final larval stage and the onset of the pupal stage before these cells initiate apoptosis (Fig. 1a). We have chosen to analyse PG samples from day 0 and day 6 of the 5th instar of *Bombyx mori* because there are striking differences in the hormonal milieu in these two developmental time points. On day 0, PG cells secrete very low amounts of ecdysteroids while the juvenile hormone titre is high^24,25^, whereas on day 6 PG cells secrete high amounts of ecdysteroids while the juvenile hormone titre is low^24,25^ while in both days the PG cells are not fully stimulated by prothoracicotropic hormone^25^. In addition, the onset of wandering behaviour on day 6 is the safest benchmark that the animals have been developing orderly and there has been extensive research carried out on the signalling pathways of PG cells of *Bombyx mori* on day 6.

We identified the receptors that participate in these cascades, determined their expression profile during this developmental stage and then illustrated and linked the expression of these receptors to KEGG database signalling pathways^23,26^ present in PG cells (Fig. 2). The datasets presented in this study were generated from PG cells from day 0 and day 6 (onset of wandering stage) of the final larval instar, the 5th instar (Fig. 1a,b). A set of 3 biological replicates of PG samples from day 0, and 3 biological replicates of PG samples from day 6 were subjected to proteomic analysis by liquid chromatography-tandem mass spectrometry (LC-MS/MS) and an identical set was subjected to transcriptomic (RNA-seq) analysis (Fig. 1).

To isolate the PGs, larvae were anesthetized by submersion in water and the two PGs of each larva were dissected rapidly (~2 min/animal) in sterile saline (0.85% NaCl). For LC-MS/MS analysis, following pre-incubation for 15**–**30 min in Grace’s medium (Invitrogen), glands were meticulously cleared of any associated tissue or debris, pooled and successively transferred to gradually diminishing volumes of Grace’s medium drops (*n*=5) before being snap frozen in dry ice and stored at −80 °C before further processing.

For RNA-seq analysis, total RNA was isolated from PGs from day 0 and day 6 of the 5th instar as described below. PGs were meticulously cleared of any associated tissue or debris and total RNA was immediately extracted with TRIzol (Invitrogen) according to the manufacturer’s instructions. The lllumina mRNA-Seq Sample Prep Kit was used according to the manufacturer’s instructions (1,004,898 Rev.D). Briefly, using oligo-dT magnetic beads, mRNA was isolated from total RNA and after mRNA fragmentation, cDNA was synthesised, ligated with sequencing adapters and amplified by PCR. Quality and yield after sample preparation was measured with the Agilent 2,100 Bioanalyzer (Agilent Technologies). Resulting products size distribution had a broad peak between 200–500 bp on a DNA 1,000 chip. Next, 17 pM of DNA was used for clustering and DNA sequencing on lllumina cBot and HiSeq2500 (HCS v2.2.58 software) according to the manufacturer’s protocols.

For qPCR, total RNA was isolated from PGs from each day of the 5th instar and the 1st day of the pupal stage (P−0; Fig. 1) as described above (*n*=7 for each day of the investigated developmental stage). Using 200 U Superscript III reverse transcriptase (Invitrogen) in 20 μl reaction volumes, first strand cDNA was synthesized from 2 μg total RNA with an oligo(dT)_20_ primer (Invitrogen) according to manufacturer’s instructions (*n*=7 for each day of the investigated developmental stage) and used in qPCR. MIQE guidelines-adopted^27^ complete protocols of the quantitative PCR are fully described in our supporting paper^20^.

### Proteomic analysis of *Bombyx mori* PG cells

Prothoracic glands were resuspended in 150 μl lysis buffer containing 100 mM Tris-HCl, pH 7.6, 4% SDS and freshly made 100 mM DTT. Samples were incubated for 3 min at 95 °C, followed by 20 min incubation in a sonication water bath and then centrifuged at 17,000×g for 30 min at 4 °C. Protein extracts were processed according to the Filter Aided Sample Preparation (FASP) protocol^28^ using spin filter devices with 10kDa cutoff (Sartorius, VN01H02). The 150 μl lysate was diluted in 8 M Urea/100 mM Tris-HCl pH 8.5, the filters were extensively washed with the urea solution, treated with 10 mgml^−1^ iodoacetamide in the urea solution and incubated for 30 min in the dark for cysteine alkylation. Proteins on the top of the filters were washed three times with 50 mM ammonium bicarbonate and finally digested by adding 1 μg trypsin/LysC mix in 80 μl of 50 mM ammonium bicarbonate solution (Mass spec grade, Promega) and incubated overnight at 37 °C. Peptides were eluted by centrifugation and upon speed-vac-assisted solvent removal eluted peptides were reconstituted in 0.1% formic acid, 2% acetonitrile in water and transferred into glass sample vials. Peptide concentration was determined by nanodrop absorbance measurement at 280 nm and 2.5 μg peptides were pre-concentrated with a flow of 3 μlmin^−1^ for 10 min using a C18 trap column (Acclaim PepMap100, 100 μm×2 cm, Thermo Scientific) and then loaded onto a 50 cm C18 column (75 μm ID, particle size 2 μm, 100 Å, Acclaim PepMap RSLC, Thermo Scientific). The binary pumps of the HPLC (RSLCnano, Thermo Scientific) consisted of solution A (2% (v/v) acetonitrile in 0.1% (v/v) formic acid) and solution B (80% acetonitrile in 0.1% formic acid). The peptides were separated using a linear gradient of 4% solution B up to 40% in 450 min for an 8 h gradient run with a flow rate of 300 nl/min. The column was placed in an oven operating at 35 °C. For LC-MS/MS, purified peptides were analysed by HPLC MS/MS coupled to an LTQ Orbitrap XL Mass spectrometer (Thermo Fisher Scientific, Waltham, MA, USA) equipped with a nanospray source. Full scan MS spectra were acquired in the orbitrap (*m*/*z* 300–1,600) in profile mode and data-dependent acquisition, with the resolution set to 60,000 at *m*/*z* 400 and automatic gain control target at 10^6^ ions. The six most intense ions were sequentially isolated for collision-induced (CID) MS/MS fragmentation and detection in the linear ion trap. Dynamic exclusion was set to 1 min and activated for 90 s. Ions with single charge states were excluded. Lock mass of m/z 445, 120025 was used for internal calibration. Xcalibur (Thermo Scientific) was used to control the system and acquire the raw files. Peptides were identified using the Proteome Discoverer 1.4 software (Thermo Scientific). The Orbitrap raw data (Data Citation 3), with peak S/N threshold set to 1.5, were searched using SEQUEST HT against the Uniprot *Bombyx mori* entries (14,788 sequences; Data Citation 5) with strict trypsin specificity and with maximum two missed cleavages and variable modifications of methionine oxidation, deamidation of glutamine and asparagine residues and acetylation of the N-terminus. Carbamidomethylation of cysteines was set as static modification. The identified peptides were filtered based on their Xcorr values versus peptide charge states (XCorr >2 for charge state +2 and XCorr >2.5 for charge state +3). The raw results without any further processing to remove false positives and duplicate entries are presented in Data Citation 5.

### The *Bombyx mori* reference genome

For our analyses, a *Bombyx mori* reference genome was assembled^20^ (Data Citation 4) by incorporating assembled scaffolds anchored to chromosomes (http://sgp.dna.affrc.go.jp/pubdata/genomicsequences.html) and genome contigs assembled to scaffolds, but not anchored to chromosomes. Scaffolds that were less than 20 kb in length were excluded. The remaining sequences (chromosomes and scaffolds) were merged to a final FASTA file which was used to construct a Bowtie2^29^ (http://bowtie-bio.sourceforge.net/bowtie2/index.shtml) index for subsequent use with TopHat2^30^ and Bowtie2^29^ aligners. In addition, a comprehensive gene set was constructed from genes anchored to chromosomes and additional genes that were inferred in the scaffold sequences and are available in KAIKObase (http://sgp.dna.affrc.go.jp/KAIKObase/). This comprehensive gene set was used to construct a gene file in GTF format to supply it to the TopHat2^30^ aligner. The source code and all files pertinent to this analysis are included in Data Citation 4.

### Sequencing and alignment of short reads

Image analysis, base calling, and quality check were performed with the lllumina data analysis pipeline RTA v1.18.64 and Bcl2fastq v1.8.4. Reads were on average 13.26 Gb for day 0 (V−0) samples (*n*=3) and 13.69 Gb for day 6 (V−6) samples (*n*=3) (Data Citation 2), 90.6% of clusters passed lllumina filters^27^ and percentage of bases with Q-score ≥30 were 89.36%. The resulting FASTQ files containing pair-end 125 bp sequence reads were subjected to quality control using the FastQC^31^ package and mapped on the reference genome using TopHat2^30^ with the standard parameters for reads obtained with Illumina platforms apart from the following:

• the *--GTF* parameter was supplied with additional transcript annotation data for the *Bombyx mori* reference genome as described above.
• the *--mate-inner-dist* and *--mate-std-dev* parameters which are crucial for paired-end reads were estimated from the Bioanalyzer reports provided from the sequencing of each sample.
• *--read-gap-length* and *--read-edit-dist* were set to 3 (default is 2) to allow some more freedom in the strictness of the overall alignment procedure since the *Bombyx mori* genome is not fully annotated yet.

After completing a first round of spliced alignment with TopHat2, reads which failed to map to the reference genome were supplied to the *bedtools bamtofastq* command from the BEDTools suite (https://github.com/arq5x/bedtools2) to create a FASTQ subset of the original raw short reads. These short reads subset was subjected to a second round of unspliced alignment with Bowtie2 in sensitive mode (options applied: *--local --very-sensitive-local --maxins 1,000 --dovetail*) to allow mapping of part of this subset back to the reference genome. Mapping of this subset back to the reference genome occurred when a sufficient and continuous proportion of each read (first 50 bases) was successfully aligned. This procedure allowed for the alignment of paired-end reads located quite further than the average pair distance. These two rounds of alignment procedure led to increased alignment rates.

### Statistical analysis

Statistical analysis was performed using the Bioconductor package metaseqR^32^. Specifically, the BAM files, one for each RNA-seq sample, were summarized to a gene read counts table, using the Bioconductor package GenomicRanges^33^. In the final read counts table, each row represented one gene, each column one RNA-Seq sample and each cell the corresponding read counts associated with each row and column. The gene counts table was normalized for inherent systematic or experimental biases (e.g., sequencing depth, gene length) using the Bioconductor package edgeR^34^ after removing genes that had zero counts over all the RNA-seq samples (891 genes). The output of the normalization algorithm was a table with normalized counts. Prior to the statistical testing procedure, the gene read counts were filtered for possible artefacts that could affect the subsequent statistical testing procedures. Genes presenting read counts below the median read counts of the total normalized count distribution (7,139 genes with cut-off value 199 normalized read counts) were excluded from further analysis. The total number of genes excluded due to the application of gene filters was 8,051. The resulting gene counts table was subjected to differential expression analysis for the contrasts day 0, 5th instar versus day 6, 5th instar using the Bioconductor packages DESeq^35^, edgeR^34^, limma^36^, NBPSeq^37^, NOISeq^38^ and baySeq^39^. To combine the statistical significance from multiple algorithms so as to optimize the trade-off between true positives and false hits, we applied the PANDORA^32^ weighted p-value method across all results. Setting the p-value (FDR or adjusted p-value) threshold to 0.05, we identified 5,539 differentially expressed genes with statistically significant p-value and of these 928 were up-regulated, 1,077 were down-regulated and 3,534 were not differentially expressed according to an absolute fold change cut-off value of 1 in log_2_ scale.

### RNA-seq data visualisation

To create the UCSC Genome Browser visualization track data hub (http://epigenomics.fleming.gr/tracks/hs_trackhubs/ekpa_dedos_2/hub.txt), BAM files resulting from the alignment procedure were converted to BED format (https://genome.ucsc.edu/FAQ/FAQformat.html#format1) using the *bedtools bamtobed* command from the BEDTools suite (http://bedtools.readthedocs.org/en/latest/index.html#) with the *-split* option to report RNA-seq reads split by the TopHat2 algorithm as separate alignments, referred hereafter as ‘tags’. The RNA signal from these files was extracted by reformatting them in BedGraph (http://genome.ucsc.edu/goldenPath/help/bedgraph.html) format using the *bedtools genomecov* command from the BEDTools suite with the *-bg* option and then to bigwig (https://genome.ucsc.edu/goldenpath/help/bigWig.html) format using the *bedGraphToBigWig* program supplied by UCSC. The bigWig tracks were visualized in a custom UCSC Genome Browser track data hub, hosting the *Bombyx mori* (bmori2) reference genome and the normalized (total signal of 10^10^) RNA-seq samples. The track data hub is available at http://epigenomics.fleming.gr/tracks/hs_trackhubs/ekpa_dedos_2/hub.txt and this link must be pasted in the My Hubs tab of the UCSC Genome Browser application.

### Signalling pathway analysis and annotation through KEGG database

KEGG pathway maps (http://www.genome.ad.jp/kegg/pathway.html) where available for the silkworm *Bombyx mori*^23,26^ or the fruit fly *Drosophila melanogaster*^23^ were used to identify proteins that are components of each pathway and examine the presence of each protein in our LC-MS/MS datasets (Data Citation 1,Data Citation 3 and Data Citation 5) and its coding gene expression in our RNA-seq datasets (Data Citation 1 and Data Citation 2). Each component of the map was subjected to blastp at the KAIKObase^18^ website to identify the corresponding gene in our dataset. Signalling pathway maps were manually curated to highlight the different features we identified (Fig. 2). Each of the illustrated pathways in Fig. 2 met the criterion that it had at least three of its components identified in our LC-MS/MS datasets and was not previously reported to be comprehensively present in PG cells of *Bombyx mori*.

### Code availability

The code used to analyse the RNA-seq data, described at various levels herein, is available in an archive within Data Citation 4 with further explanations in this link (http://epigenomics.fleming.gr/metaseqr_runs/HS/000004/). Briefly, the code consists of: i) Custom Perl and Linux shell scripts used to 1) create the *Bombyx mori* reference genome assembly for RNA-seq short read alignment, 2) create the GTF gene file and 3) generate files suitable for custom visualization of the UCSC Genome Browser. ii) Custom Linux shell scripts that can be used to reproduce the two rounds of alignment procedure described in ‘Methods’. iii) Custom R script that can be used to reproduce the differential expression analysis.

## Data Records

The annotated list of *Bombyx mori* genes file (Data Citation 1) is available on Figshare (doi:10.6084/m9.figshare.3420235).

The raw reads of our transcriptomic (RNA-seq) data have been deposited to the NCBI Short Read Archive (SRA, http://www.ncbi.nlm.nih.gov/sra/) under accession number SRP062258 (Data Citation 2). This record combines the 3 biological replicates from day 0 (http://www.ncbi.nlm.nih.gov/biosample/SAMN03978782) and the 3 biological replicates from day 6 (http://www.ncbi.nlm.nih.gov/biosample/SAMN03978783) presented in this study.

The mass spectrometry proteomics data have been deposited to the ProteomeXchange Consortium via the PRIDE^40^ partner repository with the dataset identifier PXD004265 and 10.6019/PXD004265 (Data Citation 3).

The *Bombyx mori* reference gene file in GTF format together with a series of files centred around the *Bombyx mori* genome (Data Citation 4) is available on Figshare (doi:10.6084/m9.figshare.3420412).

The Proteome Discoverer data analysis files (Data Citation 5) are available on Figshare (doi:10.6084/m9.figshare.342031).

## Technical Validation

### Assessment of false negative and false positive hits of *Bombyx mori* cell membrane receptors in RNA-seq and LC-MS/MS datasets

Based on the initial bioinformatic identification of 369 cell membrane receptors present in the genome of *Bombyx mori*, we analysed by qPCR the expression of 339 transcripts out of the initial 369 cell membrane receptor genes. Due to high sequence similarity between some transcripts a total of 30 genes could not be analysed by qPCR^20^. Of those 339 genes that were analysed by qPCR, 29 genes were found to be false negative, i.e., expressed in the PG cells (Table 1), mainly because their expression showed peaks on days other than day 0 (V−0) or day 6 (V−6) from which the RNA-seq and LC-MS/MS samples were derived. In 2 of the 29 instances, the RNA-seq data returned negative results due to incorrect annotation of the *Bombyx mori* genome and in 1 instance (i.e., putative prostanoid receptor; Table 1), gene *BMgn010037* is annotated as only a fragment of the full open reading frame.

The RNA-seq and LC-MS/MS data was more complicated for the false positive results, i.e., receptors that were not found to be expressed in the PG cells by qPCR assays (Table 2). These cases were 19 in total (Table 2). In 3 cases the reason for being false positives could be attributed to no longer being expressed in the 5th instar but were expressed in the 4th instar (Table 2). In 3 cases the RNA-seq reads were aligning with sequences found within introns while in 3 other cases the LC-MS/MS data returned hits incorrectly annotated in the UniProt *Bombyx mori* sequence database (14,788 sequences). In the remaining 10 cases the reason for getting negative results in qPCR assays and the RNA-seq data is probably due to a combination of LC-MS/MS analyses artefacts and incorrect annotation of the *Bombyx mori* genome.

### Quantitative PCR analysis of false negative and false positive hits of *Bombyx mori* cell membrane receptors in RNA-seq and LC-MS/MS datasets

The MIQE guidelines-adopted^27^ complete protocols of the qPCR assays are fully described in our supporting paper^20^. All primers, including those shown in Tables 1 and 2, were designed with an online tool (http://primer3plus.com/cgi-bin/dev/primer3plus.cgi) using the following custom settings: 1) amplicons size range 230–270 bp, 2) primer size minimum 18 bases, optimum 20 bases, maximum 23 bases, 3) primer *Tm* minimum 59 °C, optimum 60 °C, maximum 62 °C, maximum difference 1 °C, 4) Primer GC% minimum 30, optimal 50, maximum 80, and the other settings were left to default values. Among the various combinations of primers returned by the online tool, we selected the pair that combined the following criteria: 1) minimal penalty value, 2) amplicon at the 3′ end of the cDNA sequence, 3) unique hit in the NCBI Primer-BLAST webpage (http://www.ncbi.nlm.nih.gov/tools/primer-blast/index.cgi?LINK_LOC=BlastHome) using the RefseqmRNA database and *Bombyx mori* as the organism, 4) unique hit in the KAIKObase website using Blastn.

## Usage Notes

The annotation of KEGG pathway maps we provide in Fig. 2 can serve as a reference tool for other researchers who want to analyse the contribution of each of these signalling pathways to ecdysteroidogenesis. However, it is critical to note that all these signalling modules may not simply serve ecdysteroidogeneis, i.e., ecdysteroids secretion can not be the only readout of the activation or inhibition of components of each pathway in PG cells. The perplexing array of these pathways and the scarcity of data regarding other functions that PG cells may carry out in the insect body makes it difficult at present to assign discrete roles to each one of them and maybe there can not be a single role to be assigned to each one of them. For example, the Hedgehog signalling pathway has been linked to ecdysteroidogenesis in *Drosophila melanogaster*^13^ but does this link mean that the Wnt and the TGF signalling pathways (Fig. 2) are also involved in ecdysteroidogenesis?

Data Citation 2 contains the raw RNA-seq data from *Bombyx mori* PGs. The files have been analysed with up-to-date software packages such as TopHat2^30^ and Bowtie2^29^ aligners for short read mapping to the *Bombyx mori* reference genome, and the Bioconductor package metaseqR with the PANDORA^32^ method for normalization and statistical analyses. Our data can be used to examine the performance of other short read aligners or it can be a suitable resource in studies that map differential gene expression in various tissues or organs of this or other insect species.

Data Citation 3 contains the raw proteomics data from *Bombyx mori* PGs. The files have been analysed with the software package Proteome Discoverer 1.4. We believe that extending the data analysis to obtain quantitative protein profiles from the existing raw files using available proteomic analysis platforms would be quite interesting because it will provide a dimension of protein abundance in PG cells. Furthermore, identification and differential quantitation of post-translational modifications will be of major importance. Being datasets derived from control, untreated whole tissue extracts, our data can be a suitable resource when combined with future studies on organelle-enriched proteomic analysis or phosphoproteomic analysis of ecdysteroidogenesis in insects.

We believe that the publicly available custom track data hub resource on UCSC server (http://epigenomics.fleming.gr/tracks/hs_trackhubs/ekpa_dedos_2/hub.txt) hosting our RNA-seq data can be the appropriate visual tool in deciphering gene and exon/intron boundaries of yet uncharacterized *Bombyx mori* genes and identifying previously unidentified genes through the extensive visual inspection features of the tracks.

We encourage other research teams to compare our Proteome Discoverer version 1.4. data analysis files (Data Citation 5) with different or more up-to-date software and analyse our raw data (Data Citation 3) to yield further insight than we already report.

The annotated gene list of *Bombyx mori* that we provide here (Data Citation 1) can be integrated with the publicly available datasets on KAIKObase^18^ and can serve as a template for further annotation of *Bombyx mori* genes.

The *Bombyx mori* reference gene file in GTF format (contained within Data Citation 4) can be the appropriate resource to use in follow-up research on the expression profiles of other genes that participate in ecdysteroidogenesis. Data Citation 4 contains i) the assembled *Bombyx mori* genome as described in the ‘Methods’ section, ii) the assembled *Bombyx mori* transcriptome assembled like the *Bombyx mori* genome and used for optimizing the short read alignment procedure, iii) the assembled *Bombyx mori* genes in GFF, GTF and text tab-delimited format, iv) bowtie2 indexes for the *Bombyx mori* genome, v) bowtie2 indexes for the *Bombyx mori* transcriptome and vi) custom code used for the analysis.

With the full report produced by the Bioconductor package metaseqR^32^ (http://epigenomics.fleming.gr/metaseqr_runs/HS/000004/), researchers may explore a rich set of quality controls regarding the raw data (Data Citation 2), as well as several analytics regarding sample quality, normalization effects and differential expression analysis. In addition, researchers may download the full list of *Bombyx mori* genes coupled with RNA abundance values and other quality metrics as well as all the necessary parameters that will help them reproduce the differential expression analysis.

### Samples, subjects, and data outputs

Detailed information accounting for each sample, the data-generating assays applied to each sample and the resulting data outputs are provided in Data Citation 1.

## Additional Information

**How to cite this article:** Moulos, P. *et al.* Combinatory annotation of cell membrane receptors and signalling pathways of *Bombyx mori* prothoracic glands. *Sci. Data* 3:160073 doi: 10.1038/sdata.2016.73 (2016).

## Supplementary Material



## Figures and Tables

**Table 1:** False negative results of cell membrane receptors expressed in prothoracic glands cells of *Bombyx mori*.

**No**	**Receptor Type**	**public ID (gene)**	**chr. ID**	**chr. start position**	**chr. end position**	**Protein description**	**Identified by qPCR**	**Identified by RNA-seq**	**Identified by LC-MS/MS**	**rpgm V−0**	**rpgm V−6**	**False negative reason**	**Forward primer**	**Reverse primer**
**1**	Class A GPCR	BMgn003863	chr1	21684307	21697706	Neuropeptide receptor A18	√	—	—	0.001	0.005	Expressed on days other than V−0/V−6	ATGAAGGAGGAGATGGAGAGG	CCGAAGTTGGTGTTGAAGAAC
**2**	Class A GPCR	BMgn010037	chr7	698359	701773	Putative prostanoid receptor	√	—	—	0.033	0.017	Incorrect annotation	TGCAGTCTATGAACCACAACG	TGACACAGAGTGGAGGGAGATAC
**3**	Class A GPCR	BMgn006929	chr10	11044278	11051377	Serotonin receptor 4 (Ser-4)	√	—	—	0.019	0.003	Expressed on days other than V−0/V−6	ACGAGCCCCGAAAAACAATC	CTTCACAATCACACGTCGGAAC
**4**	Class A GPCR	BMgn011918	chr11	6234327	6250382	Serotonin receptor 1A	√	—	—	0.006	0.021	″	AAACTGCTACGTTGCAAGCG	AATCACCCCTCTCCGAATCAAC
**5**	Class A GPCR	BMgn011934	chr11	6688420	6738515	Protein trapped in endoderm-1-like/Trehalose receptor-like	√	—	—	0.003	0.019	″	CCTCAGCTGTGTCAACTTTTCG	TCGTGCACGAAAACGTGTTC
**6**	Class A GPCR	BMgn012114	chr11	17176223	17270624	Neuropeptide receptor A10	√	—	—	0.019	0.020	″	GAAGATGCGGAAAGAGAACG	TCGTTGAGCCAAGCATAGAG
**7**	Class A GPCR	BMgn010612	chr12	11968440	12002234	Diapause hormone receptor	√	—	—	0.008	0.023	″	TCAAGATGACCCTGTGCTG	TTCGGCTGTCTGGTTTGC
**8**	Class A GPCR	KAIKOGA006380	chr18	10560901	10565919	Orphan G protein-coupled receptor (moody-like)	√	—	—	0.002	0.014	No public ID	AGCTGCTTTATCCTGCCCTTAG	ATCGGCAGCAACCAAATGAC
**9**	Class A GPCR	BMgn008534	chr18	10833446	10842187	Orphan G protein-coupled receptor (moody-like)	√	—	—	0.047	0.049	Expressed on days other than V−0/V−6	GCACCGTAGACAAAAGATCCAC	ACTGCGCGTTCATTATCACG
**10**	Class A GPCR	BMgn004033	chr19	7876790	7923680	Neuropeptide receptor A3	√	—	—	0.023	0.026	″	GGCAGCCTATCTTCTATTCCAC	AAGGATCATCGGACCTGTTG
**11**	Class A GPCR	BMgn000093	chr24	6663816	6707304	Sex peptide receptor	√	—	—	0.021	0.006	″	TACATCGCACCGTTTCTGC	AACATCGCTGGAATGACCTC
**12**	Class B GPCR	BMgn000547	chr1	9487302	9507493	Neuropeptide receptor B4	√	—	—	0.0009	0.0007	″	TCCTTCCTATTTCTGGTGAACG	CTTGTGAGCAACGCTGAAAC
**13**	Class B GPCR	BMgn009926	chr8	18797170	18877387	Neuropeptide receptor B1	√	—	—	0.004	0.006	″	ACAAGGAATGCACTGCGAAC	TTGTTCACCGCAAACGAAC
**14**	Class B GPCR	BMgn012453	chr9	9527611	9546519	Neuropeptide receptor B3	√	—	—	0.005	0.010	″	AGGCCGAACACAACAGATTG	TTCTACCGGAACTAGGGTCAAC
**15**	Unclassified GPCR	BMgn010416	chr12	5128218	5148676	Progestin and adiponectin receptor family member 3-like	√	—	—	0.028	0.022	″	GTTGCTCGCCATATACACATCG	TATCACACGGGGGAAGAACATC
**16**	RTK	BMgn005972	chr4	5940625	5947929	Receptor tyrosine-protein kinase Ror-like	√	—	—	0.001	0.0006	″	TTTGCTTGCGAGTTGATGCG	CGGGCTCATGATTTTCATCACC
**17**	RTK	BMgn003800	chr4	20352346	20366934	Venus kinase receptor 2	√	—	—	0.036	0.028	″	AAACCGGACTCTACGACTGAG	TTGGCTTTCGTTGCGTCTTC
**18**	RTK	BMgn002597	chr5	5719821	5726335	Receptor tyrosine kinase (RYK) Dnt-like-2 (Doughnut)	√	—	—	0.016	0.045	″	GTTAGCATTGAGGACCAAACGG	ATCCACTATGCAGTTCCTAGCG
**19**	RTK	BMgn008036	chr9	64319	75212	Receptor tyrosine kinase Cad96Ca-like	√	—	—	0.008	0.006	″	ACCCTGAAAGAGAATGCCTCAG	AGATCACGCGAGGTAAGGAAC
**20**	RTK	BMgn000354	chr22	10332224	10334147	Neurospecific receptor kinase (Nrk/HOP)	√	—	—	NA	NA	Incorrect annotation	CACCGTGAAGATCTACGATTGC	TCCACCTTACTGGGATTGCATC
**21**	RTK	BMgn010869	chr22	23167125	23172280	Receptor tyrosine kinase Cad96Ca-like 2	√	—	—	0.001	0.0009	Expressed on days other than V−0/V−6	GTGTTGGCAGGATTGGGTTTC	TTGGCCCAGTAACGTCTGTAG
**22**	RTK	BMgn011535	chr23	10106580	10130411	Proto-oncogene tyrosine kinase ROS-like (Sevenless)	√	—	—	0.002	0.006	″	TGGTATGAGGAAACGGGTCATC	ATTTGCAGCCACTCATGTCG
**23**	RTSK	BMgn009697	chr2	4329363	4343148	Similar to wishful thinking	√	—	—	0.039	0.031	″	CAGGGCCAGTATCAATTTGGAG	AGCGGTTGTGGTTCTTGTTG
**24**	RTGC	BMgn006801	chr10	10291050	10306675	Receptor type guanylate cyclase 32E-like isoform	√	—	—	0.014	0.020	″	CGGCGAAGAGTTGTTTCGTC	TCAGCACGTGACCAATAAATGC
**25**	Other cell membrane receptors	BMgn002138	chr1	3765095	3884662	Furrowed homologue	√	—	—	0.008	0.007	″	GTTTGCAATAGGGACGGCAAG	TTGGCAATTTCCCGCAATCG
**26**	Other cell membrane receptors	BMgn002495	chr9	14540336	14576082	Similar to lipocalin-1 interacting membrane receptor (limr)	√	—	—	0.023	0.024	″	AAAGAAGAATGCCGCATCGC	ACCGCTTTGATCCCGATGAG
**27**	Other cell membrane receptors	BMgn006572	chr10	5638905	5646813	Patched domain-containing protein 3-like	√	—	—	0.005	0.017	″	AAATGACAACGGACGCAAGC	TGTCACTGGTTTTGCGTGTTG
**28**	Other cell membrane receptors	BMgn007929	chr15	2350150	2409987	Similar to Notch	√	—	—	0.003	0.023	″	AACTGTGCGACGTCGAAATG	AACCTTTGGCGCATTGACAC
**29**	Other cell membrane receptors	BMgn008552	chr18	11888109	11902975	Interference hedgehog	√	—	—	0.017	0.013	″	GGAAACGCCGAAAACTTTGC	TATGTGGGGAGGGATTTCTTGC
The 29 genes presented in this table were not identified by both our transcriptomic (RNA-seq) and proteomic (LC-MS/MS) analysis and conclusive evidence of their expression came from the quantitative PCR (qPCR) assays using the indicated primers. Abbreviations: GPCR: G protein-coupled receptor; RTK: receptor tyrosine kinase; RTSK: receptor serine/threonine kinase; RTGC: receptor type guanylate cyclase; Chr: Chromosome; rpgm: reads per gene model; V−0: Day 0 of the 5th instar; V−6: Day 6 of the 5th instar.														

**Table 2:** False positive results of cell membrane receptors that are not expressed in prothoracic glands cells of *Bombyx mori*.

**No**	**Receptor Type**	**public ID (gene)**	**chr. ID**	**chr. start position**	**chr. end position**	**Protein description**	**Identified by qPCR**	**Identified by RNA-seq**	**Identified by LC-MS/MS**	**rpgm V−0**	**rpgm V−6**	**False positive reason**	**Forward primer**	**Reverse primer**
**1**	Class A GPCR	BMgn012112	chr11	17478581	17480570	Neuropeptide receptor A7	—	—	√ (V−6)	0.0005	0	Incorrect entry in Uniprot	ACCGGCAAGAACAATGAACG	TGTCTGCATCGCCTTGTTTC
**2**	Class A GPCR	BMgn010894	chr22	21802919	21873834	Neuropeptide receptor A30	—	—	√ (V−6)	0.001	0.001	Incorrect entry in Uniprot	GTTCCGGACACACAAGTAAACG	TTCTTCGATGTGAGGACGTGAG
**3**	Class A GPCR	BMgn002244	chr26	9024859	9062392	Neuropeptide receptor A6-A	—	—	√ (V−6)	0.003	0.003	Possible LC-MS/MS artefact	AGCGGAAACCAACAGCAAAG	TTACGGCGCAACGAATTAGC
**4**	Class A GPCR	BMgn001412	chr21	12086933	12126114	Neuropeptide receptor A28/ACP receptor	—	—	√ (V−6)	0.006	0.007	Possible LC-MS/MS artefact	TTGGGCGTTCGCTTTATTGC	TTTGACGGCGGTTCCTTTTC
**5**	Class A GPCR	BMgn012539	chr9	7510910	7518997	Opsin	—	—	√ (V−0)	0.001	0.0006	Possibly expressed in the 4th instar or LC-MS/MS artefact	GACCAGCTTTAGGCAAAACTGG	AGCTTGTCAGGAATCCTTCGG
**6**	Class A GPCR	BMgn007930	chr15	2334865	2343080	Serotonin receptor (1D-like)	—	√	—	0.066	0.003	Reads aligned in intron	TCCTTCGCCGAAGCATTTTC	CCACATTTACCACGACAGCATC
**7**	Class A GPCR	BMgn004428	chr20	2030777	2152042	Neuropeptide receptor A5	—	√	—	0.187	1,840	Reads aligned in intron	ACACCGCTTTGGAAATTCGG	TCGAAGTGAAACAGCGAACG
**8**	Class B GPCR	BMgn014256	chr8	2619	12058	Metabotropic glutamate receptor 2-like	—	—	√ (V−0)	0.0002	0.0004	Possibly expressed in the 4th instar or LC-MS/MS artefact	TCCTGGTGTGTACAGTATACGC	ACCTTGTTCGCCAACTTTCG
**9**	Odorant receptor	BMgn014699	chr1	12377437	12385061	Odorant receptor (BmOr-1)	—	√	—	0.001	0.246	Reads aligned in intron	ATGTCATCACAACGCCCAAC	TCAAGGTTAAGGGTCCGTAACG
**10**	Odorant receptor	BMgn017182	chr25	11127835	11131624	Odorant receptor (BmOr-40)	—	—	√ (V−6)	0.01	0.01	Possible LC-MS/MS artefact	GTTTTGAACCCTTCGGAGGTTC	TTGTGAGTTGCCCGACATTG
**11**	RTGC	BMgn002197	chr15	19498061	19508229	Guanylate cyclase	—	—	√ (V−0)	0.0002	0.0007	Possibly expressed in the 4th instar or LC-MS/MS artefact	ATCATGCAGGAGGTCATAGTGC	GAGCATTTCGAACATGGAGTCG
**12**	Integrin	BMgn010454	chr12	2561262	2592387	Integrin alpha 3	—	—	√ (V−0)	0.001	0.007	Incorrect entry in Uniprot	AGAAGGCGCAAGAAGCTTTG	TTAACGTGTCGCCACAAAGG
**13**	Integrin	Bmgn006002	chr4	4502310	4508762	Integrin beta 2	—	—	√ (V−6)	0.0001	0.0003	Possible LC-MS/MS artefact	AAACGTGGCCGAAGATTTGG	AACCTCATTCTCGTCTGACCAC
**14**	Immunity related receptor	BMgn006244	chr6	11216171	11233908	BmToll 12	—	—	√ (V−0)(V−6)	0.002	0.002	Possible LC-MS/MS artefact	GTACACAAACGCGATCAGGTG	TTGTGTTCGTATCGCGTTGG
**15**	Immunity-related receotor	BMgn000673	chr1	9414935	9415375	BmPGRP-L6	—	√	—	0.177	0.163	Possible incorrect annotation in genome	CAGAGCCAATGTGTTCTTCGTG	TTCCGGCAAGCTTCCAATTG
**16**	Immunity related receptor	BMgn011085	chr23	7698223	7702050	BmToll 8	—	—	√ (V−0)(V−6)	0.033	0.042	Possible LC-MS/MS artefact	ACGCTTTTGTGTGCTACAGC	ATCAGTCTTTTCCGTCGGTCTC
**17**	Immunity related receptor	BMgn011216	chr23	18953868	18963731	BmToll 9–1	—	—	√ (V−6)	0.00003	0.00003	Possible LC-MS/MS artefact	ATTGCAACATCAGCGTCTGC	TCGTCAGCATCAGATAGTGGAG
**18**	Other cell membrane receptors	BMgn001256	chr13	6549595	6569328	Low density lipoprotein receptor-related protein 4 (LRPL)	—	—	√ (V−6)	0.01	0.01	Possible LC-MS/MS artefact	TTCCGTTGTGGTTGCATGAC	TTGCGCAACACGGATAACTC
**19**	Other cell membrane receptors	BMgn002138	chr1	3765095	3884662	Furrowed/Selectin/Sushi. von Willebrand factor type A. EGF and pentraxin domain-containing protein 1	—	—	√ (V−6)	0.008	0.007	Possible LC-MS/MS artefact	GTTTGCAATAGGGACGGCAAG	TTGGCAATTTCCCGCAATCG
The 19 genes presented in this table were identified by either our transcriptomic (RNA-seq) or proteomic (LC-MS/MS) analysis but the evidence of their expression were not supported by the quantitative PCR (qPCR) assays using the indicated primers. Critical to the assessment of these hits as false positives was the visalisation of the reads in the University of California. Santa Cruz (UCSC) Genome Browser (see text for details). Abbreviations: GPCR: G protein-coupled receptor; RTGC: receptor type guanylate cyclase; Chr: Chromosome; rpgm: reads per gene model; V−0: Day 0 of the 5th instar; V−6: Day 6 of the 5th instar.														
